# Efficacy and Efficiency of Cyanoacrylate Glue in Fistula-in-Ano

**DOI:** 10.7759/cureus.72701

**Published:** 2024-10-30

**Authors:** Rajat Sharma, Siddharth B Lonare, Saurabh Nagar, Sushant Badhal, Samir Anand

**Affiliations:** 1 General Surgery, Maharishi Markandeshwar Medical College and Hospital, Solan, IND; 2 General Surgery, B. J. Government Medical College and Sassoon General Hospital, Pune, IND

**Keywords:** abscess, cyanoacrylate glue, efficacy, efficiency, fistula-in-ano, inter-sphincteric, transsphincteric

## Abstract

Aims and objective: Most research on fistula-in-ano (FIA) is being done to improve surgical outcomes and reduce complications. Cyanoacrylate glue (CAG) is one of the promising options. We evaluated the efficacy and efficiency of CAG in the treatment of FIA.

Materials and methods: A cohort of 30 patients were included who underwent treatment using CAG. Each patient followed up at one, three, and six months to evaluate improvement in pain, discomfort, and recurrence. Patients with recurrence were treated with fistulectomy.

Result: The mean age of the cohort was 48.2±14.5 years, with a male-to-female ratio of 6:1, having four diabetic patients. Inter-sphincteric (16.54%) and trans-sphincteric (13.43%) fistulae were more common than extra-sphincteric (1.4%) fistula. The efficacy of CAG was 73%, and the procedure was found efficient with significant improvement in discomfort (p-value: 0.017). The recurrence rate was 27%, which occurred more in diabetic patients (p-value: 0.001) and trans-sphincteric fistula (p-value: 0.035).

Conclusion: The CAG application is a simple and safe daycare procedure. However, the incidence of discharge and relief in pain was significantly less, but it cannot be advised to every patient of FIA. A young patient without comorbidities and with inter-sphincteric low fistula can best be treated by this method.

## Introduction

Fistula-in-ano (FIA) is an intriguing problem of the anorectal region in the general population. A patient’s first visit and precise surgical management in the initial phase play a crucial step in the healing of the fistulous tract. Fistulectomy is among gold-standard procedures practiced for ages [1]. However, this process is difficult in patients with high internal opening, women who have anterior fistulae, patients who have previous anorectal surgeries, patients who already have a disturbance of continence, and those who have pre-existing risk of incontinence such as Crohn's disease, HIV and elderly [2].

The prevalence in the general population varies greatly; in various European nations, it ranges from 1.04 to 2.32 cases per 100,000 people [3,4]. In a nation such as India, the incidence rate of FIA is 8-23 per one million people, with males experiencing it at a rate of 12 per 100,000 and females at a rate of six per 100,000. The male-to-female ratio is 2:1 [5], indicating male preponderance for FIA. The first presentation generally occurs at a mean age of 40 years. However, most patients fall in the age group of 30-50 years. It was demonstrated that patients with FIA have a lower quality of life (QOL), which is worse in those having more extension of tract and recurrent illness [6].

Risk factors for FIA include obesity, diabetes, impaired immune systems, low socioeconomic position, smoking, hyperlipidemia, and a sedentary lifestyle [7]. The anal glands in the inter-sphincteric plane are the primary source of anal fistulae. In 30.0%-40.0% of instances, abscess formation in the inter-sphincteric region is a result of trauma, inflammation, or faecolith obstructing the gland's drain channels. This abscess may progress to a fistula. About 20% of the reasons include various illnesses, such as cancer, surgical trauma, AIDS, tuberculosis (TB), lymphogranuloma venerum, inflammatory bowel diseases (IBD) (ulcerative colitis, Crohn's disease), infections, and others [8,9].

For FIA management, various methods have been recommended and detailed, such as fistulectomy, fistulotomy, and the use of cutting seton. The arsenal of these therapeutic options has lately expanded to include newer procedures, including fibrin plug, anal fistula plug, fistula laser closure (FiLaCTM), ligation of inter-sphincteric fistula tract (LIFT), and video-assisted anal fistula therapeutic (VAAFT). Due to anal sphincter injury, surgical treatment of FIA has a high risk of fecal incontinence and a large chance of recurrence. The risk of incontinence is associated more with the proximal location of the internal opening in the anal canal [10].

Since the previous surgical treatments had a significant risk of recurrence and fecal incontinence, a number of innovative procedures with minimal morbidity and great patient satisfaction have been developed [11]. 

There is no link between FIA and mortality; however, there is a strong correlation with morbidity. Chronic side effects, such as fecal and gas incontinence, recurrence, and anal canal stenosis, affect patients after therapy. The anatomic classification and surgical technique of the FIA will determine whether it will recur or not.

Fibrin glue was first introduced in the 1990s and was thought of as a good idea for closing fistula tracts since, acting as an occlusive substance, it has a low risk of incontinence because it spares sphincteric damage. Fibrin glue showed a lot of promise for successfully sealing anorectal fistulae with few problems, but newer data reflect that the success rate had dropped from 80% in the early years to less than 50% [12,13]. Fibrin glue is costly, requires trained personnel for preparation, and must be used shortly after being prepared, typically within a few hours, due to its limited shelf life and stability constraints. These factors have prevented fibrin glue from being widely used. However, fibrin glue requires an expert to prepare it and is thus not popular [14].

There are four types of tissue adhesives, which are biomimetic, synthetic, natural protein-based, and hybrid. N-butyl 2-cyanoacrylate (n-BCA), octyl cyanoacrylate (OCA), and 2-octyl cyanoacrylate (2-OCA) are the different types of cyanoacrylate glue. Cyano helps in binding protein, acrylate helps in polymerization, and a combination of both leads to tissue healing. The term "cyanoacrylate" is based on the cyano, ethylene, and various alkyl groups [15].

A variety of techniques are being applied these days to treat anorectal fistulae, but none of them are without drawbacks, including incomplete healing, large perianal incisions or scars, incontinence, and recurrence [1]. CAG could obviate problems related to fibrin glue because it is more economically available in vials with a long shelf life and does not need prior preparation [16]. CAG was first used in surgery in the year 1959 by Coover. It belongs to a class of synthetic glue and is a good alternative to fibrin glue [16].

In many ways, cyanoacrylate glue appears to be another good alternative cure for FIA. The sphincteric anatomy and musculature are unaffected by the simple, cost-effective, straightforward, repeatable, and can be a daycare procedure. When cyanoacrylate glue is injected into a fistula, the glue plugs the fistula tract, promotes fibroblast migration and activation, and improves the deposition of collagen [5].

Thus, CAG is an attractive option compared to fibrin glue and other contemporary treatments. By effectively sealing the fistulous tract and promoting tissue healing without damaging the anal sphincter, CAG minimizes the risk of fecal incontinence and recurrence, addressing the critical concerns associated with FIA surgeries.

The current study aims to evaluate the efficacy and efficiency of cyanoacrylate glue in treating FIA, with the potential to enhance patient care and quality of life for those affected by this challenging condition.

## Materials and methods

This observational, prospective cohort study was conducted on 30 patients in the Department of General Surgery at Maharishi Markandeshwar Medical College and Hospital, Kumarhatti-Solan, Himachal Pradesh, from January 2023 to May 2024.

Study design and setting

The study design was a prospective cohort study with data collected from January 2023 to May 2024. Baseline information was collected before the intervention, including patient demographics, comorbidities, and fistula characteristics using MRI and clinical assessments. During the intervention, procedural details were documented, such as the type of anesthesia used and any immediate complications.

Post-intervention follow-up was conducted at one, three, and six months to assess outcomes such as pain, discomfort, recurrence, and any postoperative complications. Data were gathered through patient interviews and clinical examinations using standardized tools, such as the visual analog scale for pain. The need for additional interventions, such as a second glue application, was also recorded during these follow-ups.

Study population

This prospective study included 30 patients who presented with uncomplicated, low-tract FIA. Eligible participants were adults between 20 and 40 years of age; however, individuals older than 40 years were also considered if they met the inclusion criteria. Patients were selected based on a comprehensive clinical history and were managed on a day-care basis to minimize hospitalization. All patients underwent magnetic resonance imaging (MRI) for definitive diagnosis and detailed mapping of the fistulous tract.

Patients eligible for inclusion were required to have an uncomplicated, low FIA, defined as a simple inter-sphincteric or low trans-sphincteric fistula without abscess formation or multiple secondary tracts. Additionally, patients had to provide informed consent and agree to adhere to the scheduled follow-up assessments.

The exclusion criteria included complex fistulae, such as supra-sphincteric or extra-sphincteric fistulae, as well as those associated with abscesses, horseshoe tracts, or secondary extensions. Patients with multiple fistulae, Crohn’s disease, rectovaginal fistulae, carcinoma of the anorectum, portal hypertension, or rectal varices were also excluded from the study.

Preoperative assessment

Patients presenting with perianal discharge or swelling associated with pain were examined. Preoperative assessment included a detailed history, digital rectal examination (DRE), and imaging (MRI fistulogram) to confirm the fistula's internal opening, tract length, direction, and type.

Relevant laboratory investigations (CBC, LFT, RFT, PT/INR, BT/CT, and RBS) were conducted. A proctoscopy was performed to assess the presence of hemorrhoids, polyps, or ulcerations and to confirm the internal fistula opening.

Imaging

MRI was performed using a 3 Tesla MRI machine. T2-weighted images were utilized to identify the fibrous wall of the fistula and differentiate between internal and external sphincters. Gadolinium-enhanced images were employed to detect active tracts and assess for abscesses or scarring.

Surgical procedure

The surgical procedure was performed under spinal anesthesia or sedation, with patients placed in the lithotomy position. Both the internal and external fistula openings were identified. Curettage and irrigation were performed using normal saline and povidone-iodine. Cyanoacrylate glue (CAG) was injected through a feeding tube carefully inserted along the entire length of the fistulous tract, from the internal to the external opening. This technique ensured even distribution of the glue throughout the tract. Proper filling of the entire tract was essential to achieve complete sealing and promote healing while minimizing the risk of recurrence. The glue polymerized within 60 seconds, and patients were discharged after two hours. The timing of the second application of CAG was based on the clinical status of each patient during follow-up visits. In most cases, the second application was performed within the first three months after the initial procedure, with the goal of achieving complete closure of the fistula and preventing further complications. The second application of CAG is restricted to persistent discharge or incomplete closure of the fistulous tract without associated abscess formation.

Postoperative care and follow-up

Patients received a 10-day course of antibiotics (ciprofloxacin and metronidazole) and were advised to avoid strenuous activities for 7-14 days. Sitz baths were recommended every six hours for seven days. Follow-up visits were scheduled at one, three, and six months. A second glue treatment was administered in cases of incomplete healing after four weeks. The pain was evaluated using a visual analog scale (VAS), and treatment efficacy was assessed based on infection rates and recurrence.

Statistical analysis

Data were analyzed using Statistical Product and Service Solutions (SPSS, version 23.0; IBM SPSS Statistics for Windows, Armonk, NY) for Windows. Quantitative data were expressed as mean ± standard deviation, while qualitative data were presented as frequencies and percentages. A chi-square test was employed to compare categorical variables, and analysis of variance (ANOVA) was used for intra-group variation. A p-value of <0.05 was considered statistically significant.

Ethical considerations

Informed consent was obtained from all participants, and the study protocol was approved by the Institutional Ethical Committee.

## Results

The study was conducted in the Department of General Surgery at Maharishi Markandeshwar University, Kumarhatti, Solan. After applying the inclusion and exclusion criteria, 30 eligible patients with FIA were enrolled. The following key aspects were evaluated:

Demographic Distribution of Patients 

Age: Among the total 30 patients, the majority of 10 (33.3%) patients were in the 41-50 year age group, followed by six (20.0%) patients each in the age groups of 51-60 years and over 60 years and four (13.3%) patients each in the age groups of 21-30 years and 31-40 years, respectively. The age range varied from 21 to 75 years, with a mean age of 48.2±14.5 years (Table 1).

**Table 1:** Distribution of studied patients based on age group

Age group	No. of patients (n=30)	Percentage
21-30 year	4	13.3%
31-40 year	4	13.3%
41-50 year	10	33.3%
51-60 year	6	20.0%
>60 year	6	20.0%
Mean Age (in years)	48.2±14.5 (range 21-75 years)	

Gender: The majority of 25 (83.3%) patients were male, and five (16.7%) patients were female. The male-to-female ratio was 6.1 (Table 2).

**Table 2:** Distribution of studied patients based on gender

Gender	No. of patients (n=30)	Percentage
Male	25	83.3%
Female	5	16.7%

Comorbidities: Among the total patients, four (13.3%) patients had type 2 diabetes mellitus (T2DM), while the majority (26, 86.7%) patients did not have any comorbidities (Table 3).

**Table 3:** Distribution of studied patients based on comorbidities

Comorbidities	Number (n=30) of patients	Percentage
T2DM	4	13.3 %
None	26	86.7 %

Demographic Distribution of Patients Based on Types of Fistulae

Digital rectal examination and probe examination: Out of 30 patients, all patients (100%) presented with external opening, and 29 patients (96.7%) had internal opening (Table 4).

**Table 4:** Digital rectal examination and probe examination

		No. of patients (n=30)	Percentage
External Opening	Present	30	100.0%
	Absent	0	0.0%
Internal Opening	Present	29	96.7%
	Absent	1	3.3%

According to the opening: Among the total 30 patients, the majority (29, 96.7%) presented with communicating tracts, which functioned as fistula. In contrast, only one patient (3.3%) had a blind sinus tract, which does not have a connection to the external surface or another internal structure (Table 5).

**Table 5:** Distribution of studied patients based on sinus tract

Sinus Tract	No. of patients (n=30)	Percentage
Blind	1	3.3%
Communicating	29	96.7%

Site and length of the opening: External opening was present in all 30 patients; the mean distance of external opening from the anal verge is 3.3±1.48 cm away from the anal verge, and the internal opening was present at 1.5±0.55 cm away from the anal verge and present in 29 patients. The mean length of the tract was 5.5±2.12 cm (Table 6).

**Table 6:** Site and length of the opening

Site	Mean Distance (cm)
External (n=30)	3.3±1.48
Internal (n=29)	1.5±0.55
Length of tract	5.5±2.12

Findings on MRI: Among the total 30 patients, the majority (16, 53.3%) patients had inter-sphincteric fistulae, and 13 (43.3%) patients were diagnosed with trans-sphincteric based on MRI findings. None of the patients presented with supra-sphincteric fistulae. Similarly, extra-sphincteric fistulae were observed in only one (3.3%) patient. All patients had external openings. Internal openings were present in the majority (29, 96.7%) patients, with only one patient (3.3%) lacking an internal opening (Table 7).

**Table 7:** Distribution of studied patients based on types of fistulae on MRI

Types of fistulae on MRI		No. (n=30) of patients	Percentage
Inter Sphincteric	Present	16	53.3%
	Absent	14	46.7%
Trans Sphincteric	Present	13	43.3%
	Absent	17	56.7%
Supra Sphincteric	Present	0	0.0%
	Absent	30	100.0%
Extra Sphincteric	Present	1	3.3%
	Absent	29	96.7%

Post-operative Findings

Immediately after surgery: Among the total cohort of patients, consisting of 30 individuals, a majority (24, 80.0%) patients reported experiencing discomfort immediately after surgery, while the remaining six (20.0%) did not. Similarly, 20 (66.7%) patients reported experiencing pain, whereas 10 (33.3%) did not report pain post-surgery. The mean pain score was 4.68±1.15, ranging from 2 to 6 (Table 8).

**Table 8:** Postoperative pain and discomfort assessment on postoperative day zero (POD-0) among study participants The pain was assessed using the visual analog scale (VAS), which ranges from 0 to 10, where 0 indicates no pain and 10 represents the worst pain imaginable. On postoperative day zero (POD-0), 66.7% of patients (20 out of 30) reported experiencing pain. The majority of these patients rated their pain between 2 and 6 on the VAS, corresponding to mild-to-moderate pain levels. The remaining 33.3% of patients (10 out of 30) reported no pain on POD-0. Discomfort refers to any non-pain-related uneasiness or physical discomfort reported by the patient, including sensations such as bloating, pressure, or uneasiness around the surgical site.

On POD-0		No. of patients (n=30)	Percentage
Discomfort	Yes	24	80.0%
	No	6	20.0%
Pain	Yes	20	66.7%
	No	10	33.3%
Mean Pain score		4.5±1.54 (range: 2-6)	

At the one-month follow-up (Table 9), nine out of 30 patients (30.0%) showed recurrence, with no further new recurrences developing during subsequent follow-ups. This means that the same eight patients with recurrence at the three-month mark were part of the initial nine identified at one month, with one patient’s symptoms improving between one and three months. The recurrence rate stabilized at eight patients (26.7%), remaining unchanged at the six-month follow-up.

**Table 9:** Follow-up after one, three, and six months

Symptom	1 Month (n=30)	3 Months (n=30)	6 Months (n=30)
Pain	53.3% (16)	36.7% (11)	26.7% (8)
Discomfort	66.7% (20)	46.7% (14)	30.0% (9)
Recurrence	30.0% (9)	26.7% (8)	26.7% (8)
Incontinence	0.0% (0)	0.0% (0)	0.0% (0)
Discharge	30.0% (9)	26.7% (8)	26.7% (8)
Blood in Stools	0.0% (0)	0.0% (0)	0.0% (0)
Pain While Defecation	0.0% (0)	0.0% (0)	0.0% (0)
Mean Pain Score	4.25±0.68	3.8±1.4	3.5±1.77
Pain Score Range	2-4	2-4	2-6

The data on pain and discomfort show gradual improvement over time. Pain was reported by 16 patients (53.3%) at one month, decreasing to 11 (36.7%) at three months, and further to eight (26.7%) at six months. Similarly, discomfort reduced from 20 patients (66.7%) at one month, to 14 (46.7%) at three months, and to nine (30.0%) at six months.

Other symptoms, including incontinence, blood in stools, and pain during defecation, remained absent throughout the study period. Discharge was reported by nine patients (30.0%) at one month, decreasing to eight patients (26.7%) at both three and six months.

The mean pain score also reflected improvement, with a reduction from 4.25±0.68 at one month to 3.8±1.4 at three months and further to 3.5±1.77 at six months, showing a decrease in pain severity over time. The range of pain scores expanded slightly from 2-4 at one and three months to 2-6 at six months.

Second Application of CAG

Among the total 30 patients, nine (30.0%) patients underwent a second application of treatment, while 21 (70.0%) patients did not (Table 10).

**Table 10:** Distribution of studied patients based on the second application of the cyanoacrylate glue

2^nd^ Application of the cyanoacrylate glue	No. of patients (n=30)	Percentage
Yes	9	30.0%
No	21	70.0%

Recurrence After Second Application of CAG

Among the total 30 patients, eight (26.7%) patients experienced a recurrence of symptoms after the second treatment application, while 22 (73.3%) patients did not (Table 11).

**Table 11:** Association of various parameters with recurrence Fisher’s exact test was used. For the "Type of fistula," two sets of p-values are presented: the first set of p-values indicates the comparison between each specific type of fistula (inter-sphincteric, trans-sphincteric, supra-sphincteric, and extra-sphincteric) and the occurrence of recurrence. The second p-value (next to the column "Type of fistula") represents the overall statistical significance when comparing all fistula types together regarding recurrence.

Parameters		Recurrence		p-value	
		Yes (n=8)	No (n=22)		
Age in years	21-30 year	0 (0.0%)	4 (18.2%)	0.027	
	31-40 year	0 (0.0%)	4 (18.2%)		
	41-50 year	1 (12.5%)	9 (40.9%)		
	51-60 year	3 (37.5%)	3 (13.6%)		
	>60 year	4 (50.0%)	2 (9.1%)		
Gender	Male	6 (75.0%)	19 (86.4%)	0.460	
	Female	2 (25.0%)	3 (13.6%)		
Comorbidities	T2DM	4 (50.0%)	0 (0.0%)	<0.001	
	No	4 (50.0%)	22 (100.0%)		
Type of fistula	Inter-sphincteric	1 (12.5%)	15 (68.2)	0.006	0.033
	Trans-sphincteric	6 (75.0%)	7 (31.8)	0.035	
	Supra-sphincteric	0 (0.0%)	0 (0.0%)	1.00	
	Extra-sphincteric	1 (12.5%)	0 (0.0%)	0.091	
Site of fistula	External opening	8 (100.0%)	22 (100.0%)	0.938	
	Internal opening	8 (100.0%)	21 (95.5%)		

There was a significant association between age, comorbidity, and type of fistula with recurrence at six months of follow-up (p<0.05) (Table 12).

**Table 12:** Distribution of studied patients based on the recurrence after the second time application of the cyanoacrylate glue

Recurrence after the 2^nd^ time application of the cyanoacrylate glue	No. of patients (n=30)	Percentage
Yes	8	26.7%
No	22	73.3%

Discomfort was found to be significantly associated with follow-ups (p<0.05) (Table 13).

**Table 13:** Comparison of various variables at follow-ups Cochran’s Q test was used.

Variables		Follow-up at 1 month	Follow-up at 3 months	Follow-up at 6 months	p-value
Pain	Yes	16	11	8	0.101
	No	14	19	22	
Discomfort	Yes	20	14	9	0.017
	No	10	16	21	
Recurrence	Yes	9	8	8	0.946
	No	21	22	22	
Incontinence	Yes	0	0	0	1.00
	No	30	30	30	
Discharge	Yes	9	8	8	0.946
	No	21	22	22	
Blood In Stools	Yes	0	0	0	1.00
	No	30	30	30	
Pain While Defecation	Yes	0	0	0	1.00
	No	30	30	30	
Mean Pain Score		4.25±0.68	3.8±1.4	3.5±1.77	0.105

## Discussion

FIA is a well-known surgical condition, historically documented since the time of Hippocrates. Recently, the use of CAG has emerged as a promising sphincter-saving treatment modality for FIA, offering advantages such as faster wound healing, early return to work, shorter hospital stays, and reduced recurrence rates. This glue has been widely used in other medical fields, including orthopedics, urology, and neurosurgery. Cyanoacrylate, particularly n-BCA, rapidly polymerizes upon contact with biological tissue, forming a strong elastic membrane that is resistant to blood and fluids. Its ease of use and rapid action make it an attractive option in surgical management [17].

Baseline and demographic parameters

Our study included 30 patients, the majority of whom were in the 41-50 years age group (33.3%). The mean age was 48.2±14.5 years, with a male predominance of 83.3%. These findings align with Barillari et al., who reported a mean age of 48.5 years and a male-to-female ratio of 26:4. Other studies, such as those by Koli et al. and Jain et al., reported younger patient populations, likely reflecting differences in demographic selection [17,18]. Type 2 diabetes mellitus was present in 13.3% of our cases, a known risk factor for poorer outcomes.

Digital rectal and probe examination

In our cohort, all patients had external openings, with 96.7% having internal openings, which is consistent with other studies. Koli et al. [17] reported that 90% of their patients had a single external opening, similar to our findings. Only one patient (3.3%) in our study presented with a blind sinus tract, which may have contributed to a better outcome due to a lower risk of infection. Barillari et al. [19] similarly reported better outcomes in patients with simpler fistula tracts.

Types of FIA

MRI findings revealed that 53.3% of our patients had inter-sphincteric fistulae, followed by 43.3% with trans-sphincteric fistulae, and only one patient (3.3%) who had an extra-sphincteric fistula. This is consistent with the findings of Kochhar et al. [20], who reported that 53.3% of patients had inter-sphincteric fistulae and 40% had trans-sphincteric fistulae.

Post-operative findings

Immediately after surgery, 80% of patients reported discomfort, and 66.7% experienced pain, with a mean pain score of 4.68±1.15. At the one-month follow-up, both pain (53.3%, p=0.02) and discomfort (66.7%, p=0.01) had significantly reduced, with a mean pain score of 4.25±0.68. By the three-month follow-up, pain and discomfort had further improved, though not significantly. At six months, 26.7% of patients still reported pain, with a mean pain score of 4.5±1.41. Discomfort was primarily defined as a sensation of a foreign body at the fistula site, which improved over time. These findings suggest that the persistence of pain may be due to the development of new abscesses, necessitating a second application of glue in some cases.

Second application

In comparison to studies such as Koli et al. [17] and Jain et al. [18], which reported high success rates after second glue applications (80-95%), our study showed limited improvement, with only one patient benefiting from a second application. This disparity may be attributed to the learning curve associated with this technique and patient selection criteria.

Recurrence

Our study observed a 26.7% recurrence rate, which is comparable to other studies, such as Zmora et al. [21], Sentowich et al. [12], and Koli et al. [17], who reported recurrence rates between 29% and 33%. The relatively high recurrence rate in our study could be attributed to improper selection of patients, as well as inadequate experience with the cyanoacrylate technique.

Limitations

The study has several limitations that must be acknowledged to provide a balanced interpretation of the findings. With only 30 patients included, the small sample size limits the statistical power of the results and affects the generalizability to broader populations. Additionally, the lack of a formal power calculation means that the sample size may not have been adequate to detect smaller, but clinically meaningful, differences.

While follow-up assessments were conducted at one, three, and six months, a longer follow-up period is necessary to evaluate the long-term recurrence and overall effectiveness of CAG treatment. Another limitation lies in the intraoperative identification of internal openings, as the use of adjuncts such as hydrogen peroxide or dyes could have improved the accuracy of the procedure. The study also identified an association between diabetes and recurrence (p=0.001), but the small proportion of diabetic patients (13.3%) limits the ability to draw definitive conclusions about this relationship.

This study was exploratory in nature, providing initial insights into the use of CAG for the management of FIA. As such, larger, multi-center randomized controlled trials are needed to confirm the findings. Additionally, variability in glue application was introduced, as the amount used was based on the length and characteristics of individual tracts, potentially influencing outcomes. Although MRI was used as a diagnostic tool, it may not be available in all healthcare settings, which could affect the reproducibility of this approach.

Lastly, the study focused solely on uncomplicated, low-tract FIA, limiting the applicability of the findings to more complex fistulae. These limitations underscore the importance of careful patient selection, technique optimization, and appropriate follow-up care to ensure the best possible outcomes.

## Conclusions

Current research in FIA management focuses on improving surgical outcomes by minimizing complications associated with traditional procedures. CAG is one of the newer, simpler, and safer techniques for treating FIA. It has been shown to be cost-effective and is associated with minimal complications. Our study demonstrated a significant reduction in patient discomfort over time, while improvement in pain was more gradual. Although discomfort decreased markedly by the six-month follow-up, pain scores showed a slower decline, reflecting the variability in patient outcomes. These findings suggest that CAG is effective in reducing discomfort and promoting healing but may require additional strategies for more rapid pain relief in certain cases.

This procedure is best suited for younger patients without comorbidities and those with inter-sphincteric low tracts. Results for trans-sphincteric and extra-sphincteric tracts were more variable. With improved expertise and larger studies, the outcomes of this procedure may further improve. However, CAG cannot be universally recommended for all types of FIA. Careful patient selection is crucial for achieving the best possible outcomes.
